# Nicotine Administration Robustly Enhances Fentanyl-Induced Dopamine Transmission within the Dorsolateral Striatum

**DOI:** 10.1523/ENEURO.0161-26.2026

**Published:** 2026-10-27

**Authors:** Rohan V. Bhimani, Sarah C. Honeycutt, Gregory Carl Loney, Jinwoo Park

**Affiliations:** ^1^Department of Biotechnical and Clinical Laboratory Sciences, University at Buffalo, The State University of New York, Buffalo, New York 14214-3005; ^2^Program in Neuroscience, University at Buffalo, The State University of New York, Buffalo, New York 14203; ^3^Behavioral Neuroscience Program, Department of Psychology, University at Buffalo, The State University of New York, Buffalo, New York 14260

## Abstract

Opioid use disorder is a prevalent public health concern in the United States. Approximately 70% of all drug overdose deaths are attributable to fentanyl, demonstrating a need to identify risk factors underlying the escalation of fentanyl use. Nicotine use is a risk factor associated with increased consumption of opioids across both clinical and preclinical populations. Our prior findings show that nicotine administration escalates consumption of opioids and that this elevated consumption persists despite adverse consequences in rodents, demonstrating a behavioral phenotype resembling those that emerge following protracted experience with opioids. Prolonged drug consumption is associated with a shift toward increased dopamine (DA) transmission within dorsolateral striatum (DLS), a brain area critical for the development of inflexible behaviors. The present study sought to characterize the behavioral and neurochemical effects of nicotine on fentanyl administration. A dose–response curve was used to determine the effect of nicotine on fentanyl consumption as a function of dose in males and females. Systemic pretreatment with nicotine-enhanced fentanyl intake across all doses relative to nicotine-naive controls, suggesting that nicotine augments responding for opioids without evident effects on sensitivity to fentanyl. Additionally, fast-scan cyclic voltammetry was applied to measure DLS DA transmission in response to fentanyl administration and to quantify any effect of nicotine on this response in male rats. Fentanyl administration enhanced DA concentrations within DLS, and this effect was potentiated by nicotine pretreatment. These findings support that enhanced DA transmission in DLS may mechanistically contribute to nicotine-induced increases in the motivation for opioids.

## Significance Statement

Misuse of synthetic opioids such as fentanyl is primarily responsible for alarming rates of fatal overdose, thus highlighting the need for studies designed to elucidate the mechanisms contributing to opioid misuse liability. Polysubstance use of opioids and nicotine-containing products is remarkably common, and multiple clinical and preclinical observations support that nicotine markedly increases the consumption of opioids. Here, using fast-scan cyclic voltammetry in rodent models, we demonstrate that fentanyl administration robustly enhances dopamine (DA) concentrations within the dorsolateral striatum, a brain area heavily implicated in multiple behavioral features of problematic drug use. Moreover, nicotine administration significantly potentiates this fentanyl-evoked DA concentration which may underlie the enhanced liability for opioid use disorders observed in users of nicotine-containing products.

## Introduction

Among misusers of opioids, polysubstance use is more common than the use of a single substance, and polysubstance use results in unique biobehavioral consequences which may exacerbate substance use disorders (Cicero et al., 2020; Compton et al., 2021; Mahoney III et al., 2021). Specifically, combined use of opioids and nicotine is particularly frequent (Frosch et al., 2000; Nahvi et al., 2006; Tuesta et al., 2011; Kohut, 2017; Weinberger et al., 2017; Wang et al., 2021). Nicotine use is predictive of increased likelihood of misusing prescription opioids (Zale et al., 2014; Yoon et al., 2015; Young-Wolff et al., 2017) and associated with increased risk for developing opioid use disorder (OUD; Parker and Weinberger, 2021) as well as suffering a fatal opioid overdose (Franklin et al., 2005). Nicotine dependence is reported in ∼85% of individuals seeking treatment for OUD (Zirakzadeh et al., 2013) with even higher rates (∼92%) estimated in nontreatment-seeking individuals (Clemmey et al., 1997; Clarke et al., 2001; Nahvi et al., 2006; Haas et al., 2008) necessitating research on the unique biobehavioral consequences of nicotine and opioid co-use.

Intravenous self-administration (IVSA) models in rodents demonstrate that nicotine administration enhances opioid consumption and that this increase is maintained despite adverse consequences (Honeycutt et al., 2022; Honeycutt et al., 2024a,b; Honeycutt et al., 2025). The inflexible seeking and taking of drugs despite adverse consequences is a phenotype characteristic of multiple substance use disorders, including OUD (Everitt and Robbins, 2005, 2013, 2016). Prolonged drug exposure promotes the eventual development of escalated and inflexible drug consumption (Gerdeman et al., 2003; Pelloux et al., 2007; Belin and Everitt, 2008; Corbit et al., 2012; Pelloux et al., 2015; Lüscher et al., 2020) similar to the rapid effects engendered by nicotine coadministration. However, the neurobiological mechanisms by which nicotine drives these effects are unclear.

Many commonly misused drugs enhance extracellular dopamine (DA) concentrations in the striatum (Di Chiara and Imperato, 1988; Koob and Bloom, 1988; Koob, 1992). Dysregulation of these dopaminergic circuits is hypothesized to underlie the expression of addictive-like phenotypes (Nestler, 2004). The emergence of escalated drug consumption and expression of inflexible drug use is correlated with changes in phasic DA release across the striatum in response to drug administration and its associated cues (Belin and Everitt, 2008). Specifically, early in drug use, drug seeking and drug-associated cues promote phasic DA release in the ventral medial striatum (VMS) particularly within the nucleus accumbens (NAc) shell; however, with extended drug experience, these same stimuli elicit increased DA transmission within the dorsal striatum, particularly the dorsolateral striatum (DLS; Takahashi et al., 2007; Willuhn et al., 2012). This shift in drug-induced DA transmission from VMS to DLS may mechanistically contribute to many of the maladaptive behaviors associated with addiction. In support, dopaminergic signaling within the dorsal striatum is thought to be critically involved in the expression of maladaptive responding to drug-associated stimuli (Everitt and Robbins, 2005, 2013, 2016), such as the formation of inflexible stimulus-response associations (Reading et al., 1991; Packard and McGaugh, 1992; Knowlton et al., 1996; Kantak et al., 2001; Featherstone and McDonald, 2004). Specifically, DLS neuronal activity is necessary for habitual instrumental responding (Yin et al., 2004, 2006; Yin et al., 2005) and DLS DA signaling originating from the substantia nigra (SN; BjoŦrklund and Lindvall, 1984; Haber et al., 2000; Reynolds et al., 2001; Faure et al., 2005) contributes to this expression (Vanderschuren et al., 2005; Belin and Everitt, 2008; Lipton et al., 2019).

Presently, the study of DA release, specifically within DLS, in response to opioids is limited (Ito et al., 2002; Palmiter, 2008) compared with that within VMS, and no published literature has examined DLS DA release in response to fentanyl and nicotine coadministration. This study addresses this gap in knowledge by applying fast-scan cyclic voltammetry (FSCV) to determine the degree to which IV fentanyl administration augments DA transmission within the DLS and how nicotine affects this transmission.

## Materials and Methods

### Animals and housing

Subjects in Experiment 1 (behavioral dose–response curve) consisted of 21 Sprague Dawley rats (male, 11; female, 10; Envigo). For FSCV procedures, only male subjects were included (*n* = 9; Charles River Laboratories). The decision to restrict these initial, highly challenging awake-behaving FSCV experiments to male rats was driven by the extreme technical demands of combining jugular vein catheterization with awake-behaving, tethered voltammetric recordings. Given the need to first establish a robust, baseline neurochemical mechanism for fentanyl at these specific fixed doses, we focused our resources on a single sex to ensure statistical power. All rats were individually housed in polycarbonate cages in a temperature and humidity-controlled facility and maintained on a 12:12 h reverse light cycle. After a 24 h acclimation to the facility, all animals were handled for 3 d prior to commencing experimental procedures. All procedures related to handling and caring for animals were performed in accordance with the “Guide for Care and Use of Laboratory Animals” (8th Edition, 2011, US National Research Council) and were approved by the Institutional Animal Care and Use Committee of the University of Buffalo.

### Surgical procedures

All rats underwent jugular catheterization in which chronically indwelling catheters were surgically implanted. Briefly, rats were anesthetized with isoflurane (1–3%) inhalation, and a 15 cm catheter line (C30PU-RJV1402, Instech Laboratories) was implanted into the right external jugular vein. The opposite end of the catheter was connected to an externalized vascular access button (VABR1B/22, Instech Laboratories) positioned just posterior to the scapular region on the dorsal side of the rat. Catheters were flushed with 0.1 ml of heparinized saline and enrofloxacin (Baytril, Bayer HealthCare) once daily for 10 d to prevent infection. Following the postoperative recovery period, catheters were flushed before and after self-administration sessions with 0.1 ml of heparinized saline. Catheter patency was verified at the conclusion of the study by administering 0.1 ml of ketamine (10 mg/ml, i.v.) and verifying the expression of ataxia.

For rats included in awake-behaving FSCV experiments, immediately following jugular catheterization, rats underwent stereotaxic surgeries for FSCV measurement. Briefly, anesthetized rats were transferred from the jugular vein surgery station and placed in a stereotaxic frame (David Kopf Instruments). The scalp was swabbed with chlorhexidine and iodine followed by a rinse with ethanol. Preincision local anesthesia was induced by a subcutaneous injection of bupivacaine (1.6 mg/kg), and a central incision was made to expose the skull. Subsequently, 1 mm holes were drilled in the skull for implantation of screws and electrodes. A guide cannula (#MD-2251, Bioanalytical Systems) for loading a micromanipulator containing a carbon-fiber microelectrode (neurochemical sensor) was implanted above the DLS [anteroposterior (AP) +0.00 mm, mediolateral (ML) 3.40 mm]. An Ag/AgCl reference electrode was implanted into the contralateral hemisphere. An untwisted bipolar, stainless-steel stimulating electrode (#MS303, Pro-Tech International) was implanted into the medial forebrain bundle [AP –4.0 mm, ML 1.4 mm, dorsoventral (DV) ∼−8.0 to 9.0 mm]. The stimulating electrode was insulated to the tip (0.2 mm diameter), and the tips were separated by ∼1.0 mm. AP, ML, and DV coordinates obtained from the rat brain atlas (Paxinos and Watson, 2006) were referenced from bregma to determine placements. All rats received postoperative care and were allowed to recover for 1 week before awake-behaving FSCV experiments.

### Chemical stimuli

Nicotine tartrate (Cayman Chemicals) was dissolved in sterile saline at a concentration of 0.4 mg/ml (freebase), and the pH was adjusted to ∼7.4 with dilute NaOH. All nicotine injections were administered subcutaneously at a dose of 0.4 mg/kg (volume of 1 ml/kg). This dose of nicotine is a standard dose used in behavioral studies conducted in rats, which is expected to produce brain concentrations of nicotine ∼21 ng/ml (Matta et al., 2007), falling within the expected range experienced by human smokers (DiFranza and Wellman, 2007; Katner et al., 2015). Fentanyl citrate (Hikma Pharmaceuticals) was diluted in sterile saline to a concentration of 8 µg/ml for IVSA dose–response procedures or 25 µg/ml for FSCV procedures.

### Operant IVSA procedures

Rats (*n* = 21; female, 10; male, 11) were assigned to receive either nicotine (0.4 mg/kg, s.c.; *n* = 11, 5 females) or vehicle (sterile saline, 0.9%, SC; *n* = 10, 5 females) at a volume of 1 ml/kg, counterbalanced across sex and bodyweight. Prior to any behavioral experiments, all rats received injections of their assigned drug (nicotine or vehicle) in their home cages once daily for 2 d in order to acclimate the animals to the injection procedures. Prior to each operant session, rats received an injection of either nicotine or vehicle according to assignment and were returned to their home cages for 15 min before being transported to the operant chambers. IVSA procedures were conducted in standard operant conditioning chambers (Med Associates) equipped with two retractable levers, a red house-light, a sound attenuating fan, and two stimulus lights located above each lever. Active and inactive levers were assigned in a counterbalanced manner across groups. Rats were trained to acquire IVSA of fentanyl citrate (1.5 µg/kg/inf) in seven daily 2 h sessions on a fixed ratio-1 (FR-1) schedule of reinforcement such that one press of the active lever resulted in retraction of both levers, illumination of the cue light corresponding with the active lever side, and delivery of an intravenous infusion of 1.5 μg/kg fentanyl. The cue light was illuminated during the entire infusion period (∼3 s). Responses on the inactive lever were recorded but resulted in no programmed consequences.

### IVSA dose–response

Following acquisition of fentanyl IVSA at the 1.5 μg/kg/inf maintenance dose, all rats were presented with various doses of fentanyl: 0.75, 1.5, 3.5, and 5.0 μg/kg/inf, administered intermittently across sessions, such that upon every other session all rats were tested with the formerly trained 1.5 μg/kg/inf dose to prevent confounding changes due to devaluation or the potential of extinction at lower doses. Variable doses were presented during alternate sessions in a semirandom order such that dose order presentation was counterbalanced across sex and nicotine/vehicle assignments using a Latin-square design. Only one dose of fentanyl was used on a given day, and each dose of fentanyl was tested once per rat.

### FSCV procedures

Glass-encased carbon-fiber and Ag/AgCl reference electrodes were prepared according to standard procedures (Cahill and Wightman, 1995; Wakabayashi et al., 2016). T-650 untreated carbon fibers (Cytec Industries), 7 µm in nominal diameter, were used and cut to an exposed length of 75–100 μm (Wakabayashi et al., 2016; Bhimani et al., 2021). For recordings, the TH-1 software (University of North Carolina Department of Chemistry Electronic Shop; Park et al., 2011; Park et al., 2015) was used to apply a triangular waveform (−0.4 V to +1.3 V and back to −0.4 V, 400 V/s, 100 ms intervals; low-pass filtered at 2 kHz) to the carbon-fiber microelectrode. Background-subtracted cyclic voltammograms were obtained by performing a digital subtraction of voltammograms collected during electrical or chemical stimulation from those collected during baseline recording and used to distinguish DA from metabolites and other neurochemicals (Heien et al., 2003; Takmakov et al., 2010; Park et al., 2011). Voltammetric responses were visualized as color plots with the abscissa as potential, the ordinate as acquisition time, and the current encoded in false color. The current was converted to concentration based on calibration curves with known concentrations of DA [10.5 ± 0.4 pA/(μM·μm^2^)]. Prior to recording procedures, rats (*n* = 9) were habituated for 2 d (1 h/day) to a custom-built open field arena (12″ × 12″ × 24″, Med Associates), equipped with a ceiling-mounted video camera, and housed within a Faraday cage to reduce electrical noise. Each rat underwent two recording sessions on consecutive days, one in which the rat received a noncontingent injection of nicotine (0.4 mg/kg, s.c.) and one in which vehicle was administered. The administration of nicotine on either the first or second recording session was counterbalanced across rats.

During FSCV recordings, a micromanipulator (University of Illinois at Chicago Research Resources Center, custom) loaded with a fresh carbon-fiber microelectrode was lowered into the DLS (−4.0 to −5.0 mm) until electrically evoked and naturally occurring phasic DA release was observed. Once electrode position was optimized in the robust DA release sites (“hotspot”), baseline recordings were conducted for 30 min. Following baseline recording, nicotine or vehicle was administered via subcutaneous injection, and recording continued for an additional 10 min. Rats then received noncontingent infusions of fentanyl (5, 5, and 15 μg/kg, i.v.), with each infusion spaced 25 min apart culminating in a total of 25 μg/kg administered per session. These doses were selected to maintain statistical power for comparisons across animals with the 5 μg/kg dose being the lowest reported reinforcing dose of fentanyl in conditioned place preference procedures (Knauss et al., 2023). Only two sessions were conducted with each rat so that we could generate reproducible measurements under both nicotine and vehicle conditions due to the fragile nature of the neurochemical sensor. At the end of all experiments, rats were deeply anesthetized with sodium pentobarbital (487.5 mg/kg) and transcardially perfused. Electrode locations were confirmed by electrolytic lesions, which were imaged using a stereo microscope (MD900, AMScope).

### Operant behavior data analyses

For operant IVSA procedures, the number of infusions earned, lever presses (active and inactive), and total fentanyl consumed during acquisition sessions and dose–response sessions were independently analyzed with mixed-measures ANOVAs. For acquisition, Drug (nicotine or saline) and Sex (male or female) served as between-subject factors, and Session served as a within-subject factor. For the dose–response curve analysis, Drug and Sex served as between-subject factors, and Dose (0.75–5.0 μg/kg) served as a within-subjects factor. Post hoc analyses were conducted with Tukey's corrections for multiple comparisons.

### FSCV data analyses

Clampfit 8.1 as part of pCLAMP 8.1 software package (Axon Instruments) was used to analyze all neurochemical data using standard procedures (Park et al., 2006). A training set was created in order to extract the DA component from a mixed analyte signal using a principal component regression algorithm (Heien et al., 2005). Electrical stimulation intensity (80–150 µA) and pulse numbers were varied to evoke different concentrations of DA and pH in the DLS at the end of experiments in order to construct a training set for chemometric analysis (Heien et al., 2005; Park et al., 2012). DA transients were considered signals when they were five five times the root-mean-square noise level (S/N > 5). Data are represented as mean ± SEM (standard error of the mean), and “*n*” values indicate the number of rats. Statistical analysis was performed using the GraphPad Software. As the effects of fentanyl on DA concentration were rapid and prevented quantification of any DA transients, baseline changes over 20 min were integrated to calculate the area under the curve (AUC) for each recording. Data were binned into 60 s averages prior to AUC analysis. These AUC values, representing overall DA concentration over the 20 min recording period, were used for subsequent statistical analyses. Cumulative AUC was used for these comparisons because DA concentrations did not return to preinjection levels between doses. A two-way ANOVA was conducted on the AUCs, and planned comparisons were conducted with Tukey's corrections for multiple comparisons at each fentanyl dose–response to compare the AUCs between nicotine- and saline-treated rats. Rats that showed damage to the sensor/sensor misplacement or excessive noise were excluded from neurochemical analysis at a specific dose(s). A total of three rats were excluded due to off-target sensor placements with no observable DA detected (*S*/*N* < 5) leaving a final total of six rats in the FSCV procedures. Statistical tables for all analyses are presented in Extended Data.

## Results

### Fentanyl IVSA acquisition

Here, rats were trained to acquire IVSA of fentanyl citrate (1.5 μg/kg/inf) across seven sessions at an FR-1 schedule of reinforcement. Rats treated with nicotine prior to operant conditioning consumed significantly more fentanyl across the 7 d acquisition phase relative to nicotine-naive controls (Fig. 1*A*). Analysis of fentanyl consumed (micrograms per kilogram) during seven sessions of acquisition in a repeated-measures ANOVA revealed that, regardless of sex or drug condition, rats increased consumption across sessions (main effect of Session; *F*_(6,102)_ = 10.19, *p* < 0.001). Nicotine administered prior to operant sessions resulted in overall greater consumption of fentanyl relative to vehicle controls regardless of sex (main effect of Nicotine; *F*_(1,17)_ = 13.35, *p* < 0.01). In general, across sessions, female rats consumed more fentanyl than males, regardless of nicotine administration (main effect of Sex; *F*_(1,17)_ = 8.87, *p* < 0.01). However, a lack of Sex × Drug interactions indicates that the degree to which nicotine-enhanced fentanyl consumption did not differ across sexes. In sum, total fentanyl consumption across the entire training period was significantly higher in nicotine-treated rats, relative to vehicle, and significantly higher in females, relative to males (Fig. 1*B*). Extended Data Figure 1-1 depicts these results disaggregated by sex. Extended Data Figure 1-2 consists of statistical tables for these analyses.

**Figure 1.**
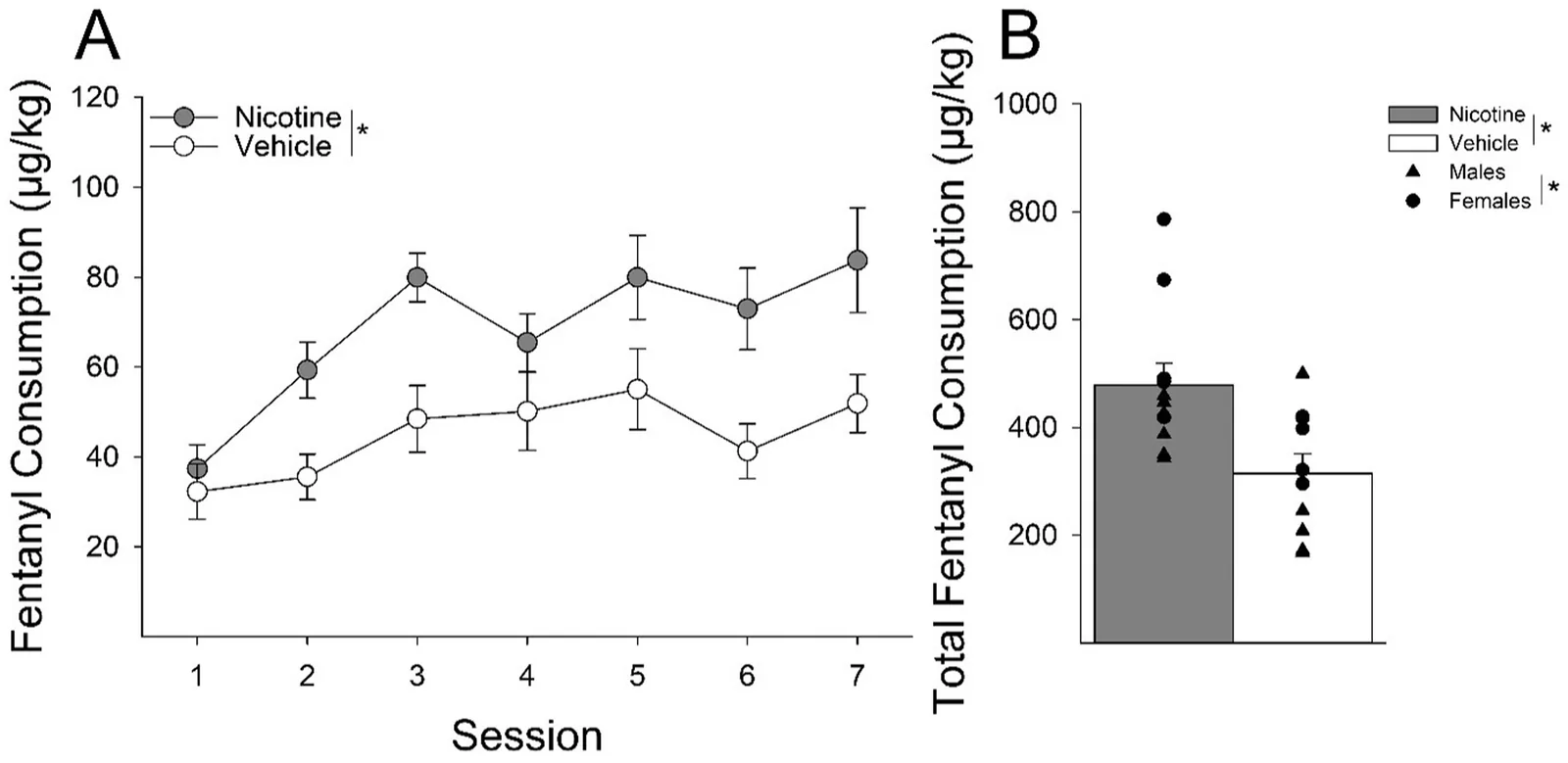
Nicotine treatment enhanced responding for, and consumption of, fentanyl compared with vehicle-treated controls across the acquisition phase. ***A***, Consumption of fentanyl (micrograms per kilogram) by nicotine-treated (*n* = 11, 5 female) and vehicle-treated (*n* = 10, 5 female) rats across 7 d of acquisition of IVSA of fentanyl (1.5 μg/kg/inf) at an FR-1 schedule of reinforcement. Nicotine-treated rats consumed more total fentanyl than vehicle-treated rats across the 7 d acquisition phase (* indicates a significant main effect of nicotine treatment of *p* < 0.01). ***B***, Total fentanyl consumption across the 7 d acquisition period was significantly higher in nicotine-treated rats compared with vehicle-treated controls (* indicates a significant main effect of nicotine treatment of *p* < 0.01). Additionally, total fentanyl consumption was significantly higher in female rats, compared with male rats, irrespective of nicotine or vehicle treatment (* indicates a significant main effect of sex of *p* < 0.01). Extended Data Figure 1-1 depicts these results disaggregated by sex. Extended Data Figure 1-2 consists of statistical tables for these analyses.

10.1523/ENEURO.0161-26.2026.f1-1 Figure 1-1 Fentanyl consumption during the acquisition of fentanyl IVSA in (A) male and (B) female rats. Download Figure 1-1, TIF file.

10.1523/ENEURO.0161-26.2026.f1-2 Figure 1-2 Statistical analyses of fentanyl consumption during acquisition as depicted in Figure 1. Download Figure 1-2, TIF file.

Rats treated with nicotine prior to operant conditioning exhibited significantly higher drug-directed responding for fentanyl relative to nicotine-naive controls (Fig. 2*A*). Analysis of percentages of accurate responding (calculated by number of active lever responses divided by total active and inactive lever responses, multiplied by 100) during acquisition revealed that, regardless of sex or drug condition, rats increased accuracy of lever responses across sessions (main effect of Session; *F*_(6,102)_ = 20.30, *p* < 0.001). Nicotine-treated rats demonstrated higher accurate lever responding across acquisition relative to vehicle controls, regardless of sex (main effect of Drug; *F*_(1,17)_ = 27.66, *p* < 0.001). Tukey's post hoc analyses revealed that nicotine-treated rats had significantly higher accuracy of active responses relative to vehicle controls on Sessions 2, 3, and 4 (Session × Drug interaction; *F*_(6,102)_ = 4.22, *p* < 0.001). By the second day of acquisition, nicotine-treated rats had reached an average accuracy of 84%, whereas vehicle controls did not reach 85% accuracy until Day 7 of acquisition. In general, female rats demonstrated higher lever accuracy earlier in acquisition training, relative to males, regardless of drug condition (main effect of Sex; *F*_(1,17)_ = 5.63, *p* < 0.05) though no sex differences were observed on the final day of acquisition training (Fig. 2*B*). As with fentanyl consumption, the effect of nicotine to increase responding for fentanyl relative to control rats did not differ across sexes. Extended Data Figure 2-1 depicts these results disaggregated by sex. Extended Data Figure 2-2 consists of statistical tables for these analyses.

**Figure 2.**
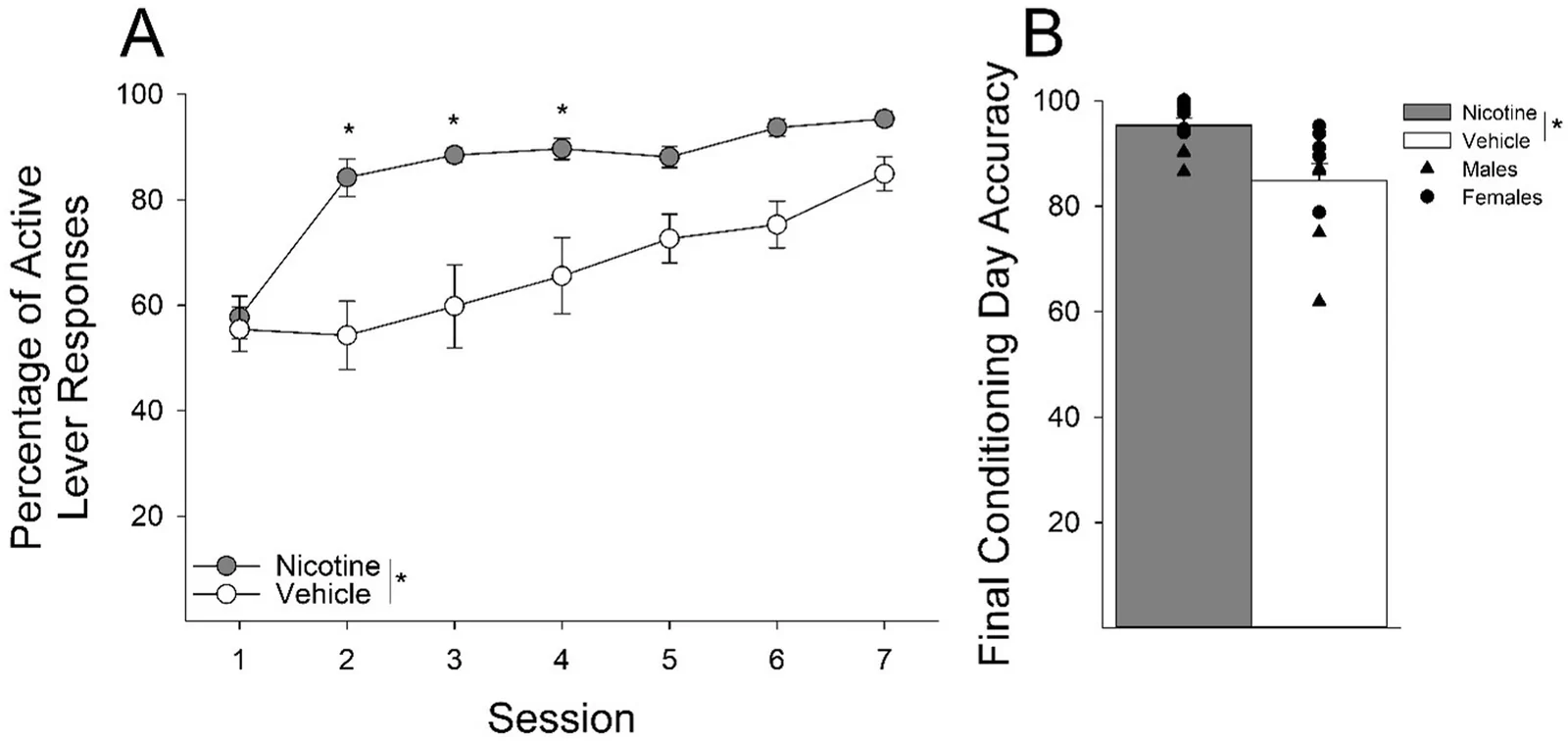
Nicotine-treated rats acquired fentanyl self-administration more rapidly than vehicle-treated controls*. **A***, The percentage of active lever responding relative to total (active + inactive) lever responses by nicotine-treated (*n* = 11, 5 female) and vehicle-treated (*n* = 10, 5 female) rats across the acquisition phase. Nicotine-treated rats exhibited higher percentages of accuracy more rapidly compared with control rats. Tukey's corrected post hoc analyses revealed that nicotine-treated rats demonstrated a higher rate of acquired drug-directed responding for fentanyl on Days 2, 3, and 4 of training (*s indicate *p*s < 0.05). ***B***, Drug-directed responding on the final day conditioning remained significantly higher in nicotine-treated, compared with vehicle-treated, rats (* indicates a significant main effect of nicotine treatment of *p* < 0.01). Importantly, all rats had acquired fentanyl self-administration by the end of training as indicated by an average active lever accuracy of 85%. Extended Data Figure 2-1 depicts these results disaggregated by sex. Extended Data Figure 2-2 consists of statistical tables for these analyses.

10.1523/ENEURO.0161-26.2026.f2-1 Figure 2-1 Percentage of accurate lever responding during the acquisition of fentanyl IVSA in (A) male and (B) female rats. Download Figure 2-1, TIF file.

10.1523/ENEURO.0161-26.2026.f2-2 Figure 2-2 Statistical analyses of the percentage of accurate responses during acquisition as depicted in Figure 2. Download Figure 2-2, TIF file.

### Fentanyl dose–response curve

Following acquisition, rats were presented with various doses of fentanyl (0.75, 1.5, 3.5, and 5.0 μg/kg/inf) intermittently across sessions. Nicotine administration resulted in a vertical shift in the dose–response curve for fentanyl relative to nicotine-naive controls (Fig. 3). Analysis of the number of fentanyl infusions earned at various doses (Fig. 3*A*) in a repeated-measures ANOVA revealed that nicotine-treated rats responded more for fentanyl compared with vehicle-treated controls irrespective of dose (main effect of Drug; *F*_(1,17)_ = 9.15, *p* < 0.01). In general, regardless of Sex or Drug condition, rats reduced responding for fentanyl as a function of the increasing dose (main effect of Dose; *F*_(3,51)_ = 39.93, *p* < 0.001). A significant Drug × Dose × Sex interaction was observed (*F*_(3,51)_ = 3.40, *p* < 0.05), and Tukey's post hoc analyses revealed this effect was driven by nicotine-treated female rats earning more infusions specifically at the lowest dose of fentanyl (0.75 μg/kg) relative to all other experimental groups (Fig. 3*C*). Extended Data Figure 3-1 consists of statistical tables for these analyses.

**Figure 3.**
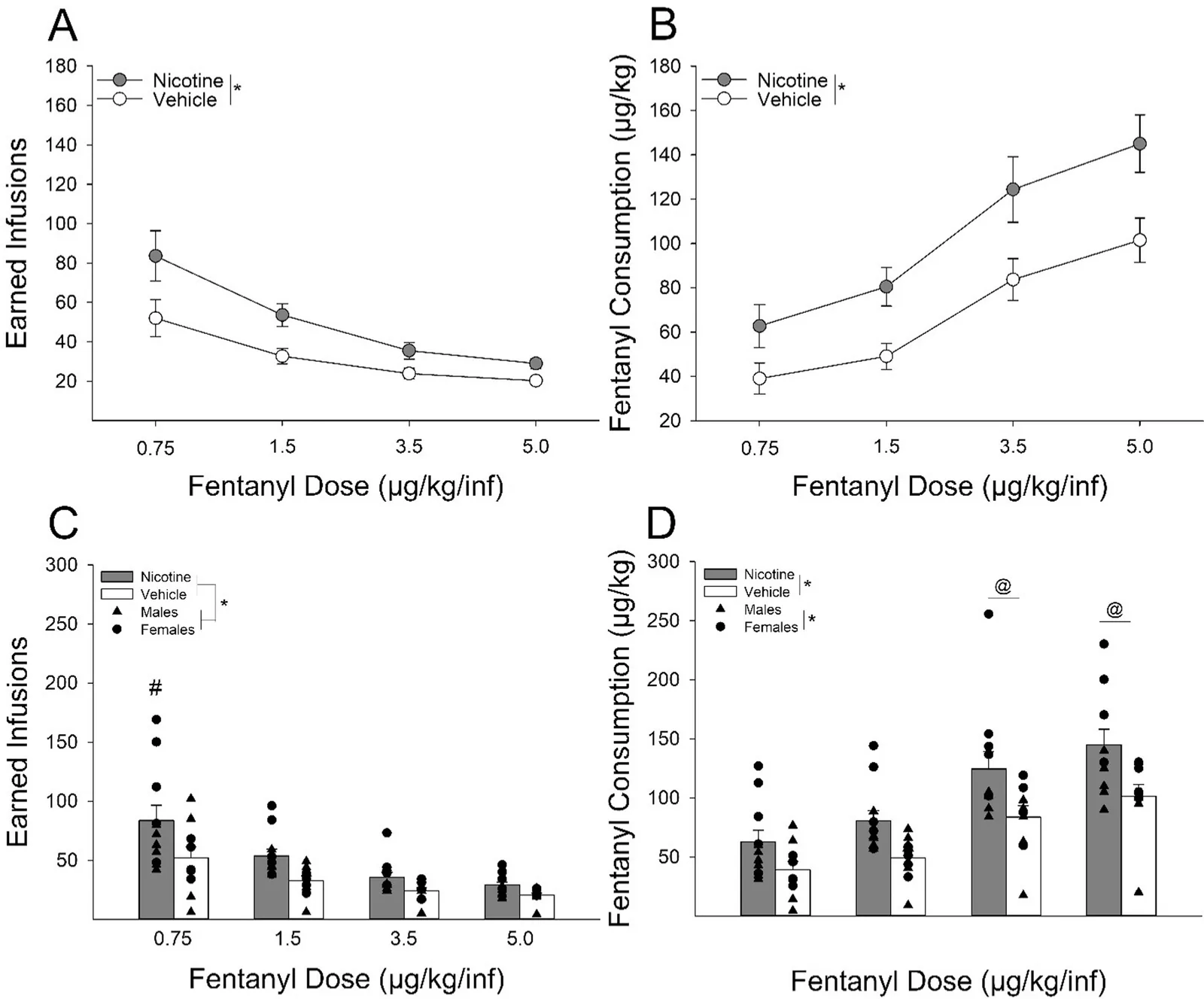
Nicotine treatment produced a vertical shift in the dose–response curve for fentanyl. ***A***, Number of earned infusions of fentanyl by nicotine-treated (*n* = 11, 5 female) and vehicle-treated (*n* = 10, 5 female) rats for various doses of fentanyl (displayed in ascending order, though order of presentation was counterbalanced between subjects). Nicotine-treated rats generally earned more infusions of fentanyl relative to control rats across all doses (* indicates a significant main effect of nicotine treatment, *p* < 0.01). ***B***, Consumption of fentanyl (micrograms per kilogram) by nicotine-treated and vehicle-treated rats at various doses of fentanyl. Nicotine treatment resulted in significantly higher consumption of fentanyl relative to vehicle treatment across all doses of fentanyl (* indicates a significant main effect of nicotine treatment, *p*s < 0.01). ***C***, There was a significant Nicotine × Dose × Sex interaction in the number of earned infusions (*p* < 0.05). Tukey's corrected post hoc comparisons revealed that this was driven by female nicotine-treated rats earning significantly more infusions at the 0.75 µg/kg dose compared with all other groups (# indicates *p* < 0.001). ***D***, There was a significant Dose × Sex interaction in the amount of fentanyl consumed (*p* < 0.01). Tukey's post hoc comparisons revealed that this was driven by female rats, regardless of nicotine or vehicle treatment, consuming more fentanyl at the two highest doses (@s indicated *p*s < 0.05). Extended Data Figures 3-1 and 3-2 consists of statistical tables for these analyses.

10.1523/ENEURO.0161-26.2026.f3-1 Figure 3-1 Statistical analyses of the number of earned infusions obtained during the dose-response curve experiments as depicted in Figure 3. Download Figure 3-1, TIF file.

10.1523/ENEURO.0161-26.2026.f3-2 Figure 3-2 Statistical analyses of the amount of fentanyl consumed during the dose-response curve experiments as depicted in Figure 3. Download Figure 3-2, TIF file.

Similarly, analysis of fentanyl consumed (micrograms per kilogram) revealed that nicotine-treated rats consumed more fentanyl across doses relative to saline-treated (main effect of Drug; *F*_(1,17)_ = 11.59, *p* < 0.01), demonstrating a vertical shift in the dose–response curve for fentanyl in nicotine-treated rats relative to saline-treated controls (Fig. 3*B*). Additionally, female rats generally consumed more fentanyl than males across doses, regardless of drug condition (Dose × Sex interaction; *F*_(3,51)_ = 5.71, *p* < 0.01), and Tukey's post hoc analyses revealed that this effect was driven by female rats consuming more fentanyl than males specifically at the two highest doses of fentanyl (3.5 and 5.0 µg/kg/inf; Fig. 3*D*). Importantly, no Drug × Dose interaction was observed, demonstrating that nicotine treatment did not differentially alter consumption of fentanyl at any particular dose relative to vehicle treatment. Extended Data Figure 3-2 consists of statistical tables for these analyses.

### Fentanyl effects on DLS DA transmission

Neurochemical sensors were vertically implanted into the DLS until reproducible DA release was observed. Once a stable baseline was recorded, rats were exposed to either saline vehicle (1.0 ml/kg, s.c.) or nicotine (0.4 mg/kg, s.c.) and then administered noncontingent, intravenous fentanyl. Representative DA concentration versus time traces at the oxidation potential for DA (∼0.65 V) in rats with saline or nicotine on board followed by fentanyl injection (5 μg/kg, i.v.) show that noncontingent delivery of fentanyl produced a rapid increase in concentration of DA in DLS, and this effect was augmented by treatment with nicotine (Fig. 4); extracted 30 s color plots show representative neurochemical changes, and the voltammogram in the inset identifies the signal as catecholamine in origin. Figure 5 shows average changes in DA concentration every 60 s 5 min before and 20 min after fentanyl administration in vehicle and nicotine-treated rats. A two-way ANOVA performed on AUC values derived from overall DA concentration over the recording period (∼20 min) following fentanyl infusion (5, 10, 25 μg/kg, i.v., escalating doses) revealed that fentanyl significantly enhanced DA concentration relative to baseline at all doses tested (main effect of Dose; *F*_(2,20)_ = 45.19, *p* < 0.001; Fig. 6) regardless of nicotine or vehicle administration. Tukey's post hoc analyses determined that 10 μg/kg significantly increased DA concentration relative to 5 μg/kg and that 25 µg/kg significantly increased above the 10 µg/kg dose (Fig. 6). Moreover, nicotine significantly potentiated fentanyl-evoked DA concentrations compared with vehicle-treated controls regardless of dose of fentanyl (main effect of Nicotine; *F*_(1,10)_ = 7.03, *p* < 0.05; Fig. 6). Planned Tukey's comparisons conducted between nicotine and saline treatments at each dose of fentanyl revealed the AUC was significantly higher for nicotine-treated rats at the 25 µg/kg (*p* < 0.05) dose of fentanyl, while the other two doses did not remain significant after correction for multiple comparisons (Fig. 6). Extended Data Figure 6-1 depicts statistical tables for these analyses.

**Figure 4.**
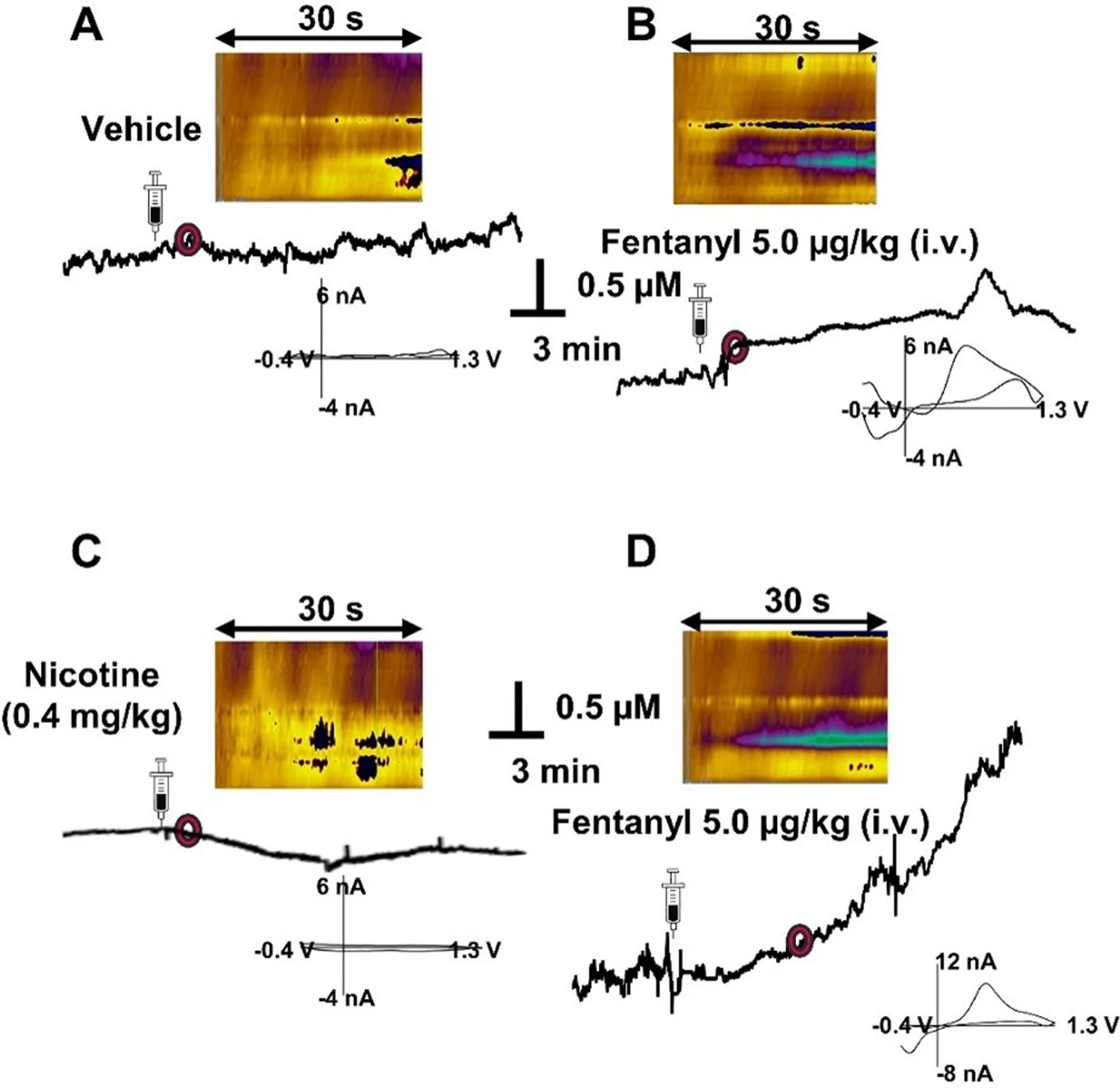
Extracted 30 s color plots of representative FSCV recording of neurochemical changes in DLS. ***A***, Representative color plot extraction (30 s) in DLS following administration of vehicle (saline, i.v.). ***B***, Representative color plot extraction (30 s) in DLS following administration of fentanyl (5 μg/kg, i.v.). ***C***, Representative color plot extraction (30 s) in DLS following administration of nicotine (0.4 mg/kg, s.c.). ***D***, Representative color plot extraction (30 s) in DLS following administration of fentanyl (5 μg/kg, i.v.) preceded (∼10 min prior) by pretreatment with nicotine (0.4 mg/kg, s.c.). Syringe icons indicate delivery of intravenous fentanyl infusion. Red circles indicate color plot extraction. Inverted red triangles denote where the cyclic voltammogram was extracted.

**Figure 5.**
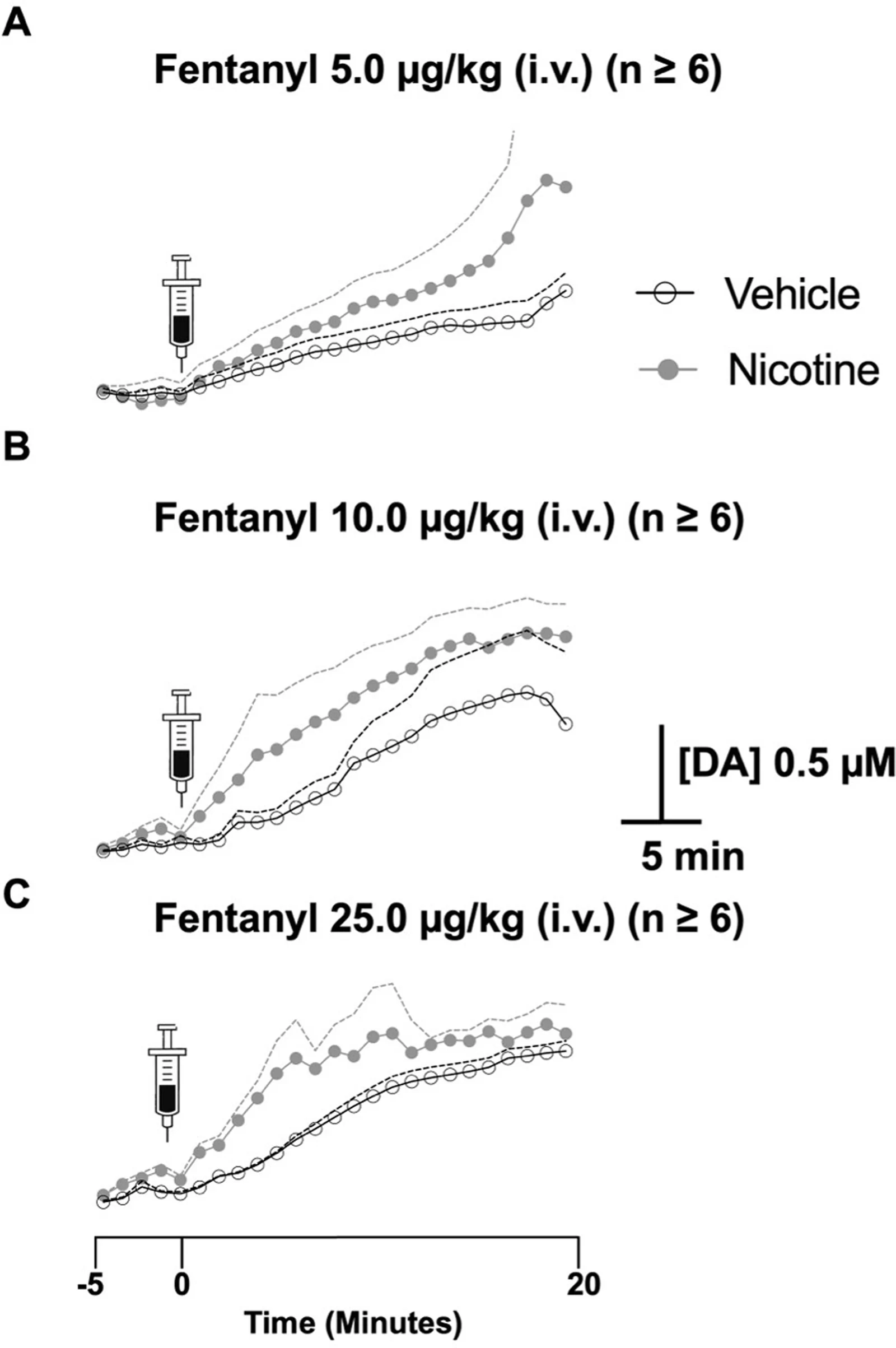
Fentanyl significantly enhanced DA concentration at all doses tested (5, 10, 25 μg/kg). Average concentrations of DA in the DLS in 60 s bins over time following administration of (***A***) 5 μg/kg, (***B***) 10 μg/kg, and (***C***) 25 μg/kg fentanyl in rats pretreated with either vehicle or nicotine (0.4 mg/kg, s.c.). Each trace is an average of six rats, SEM is indicated by the dotted lines above each trace which represent 5 min before and 20 min after fentanyl administration (indicated by syringe).

**Figure 6.**
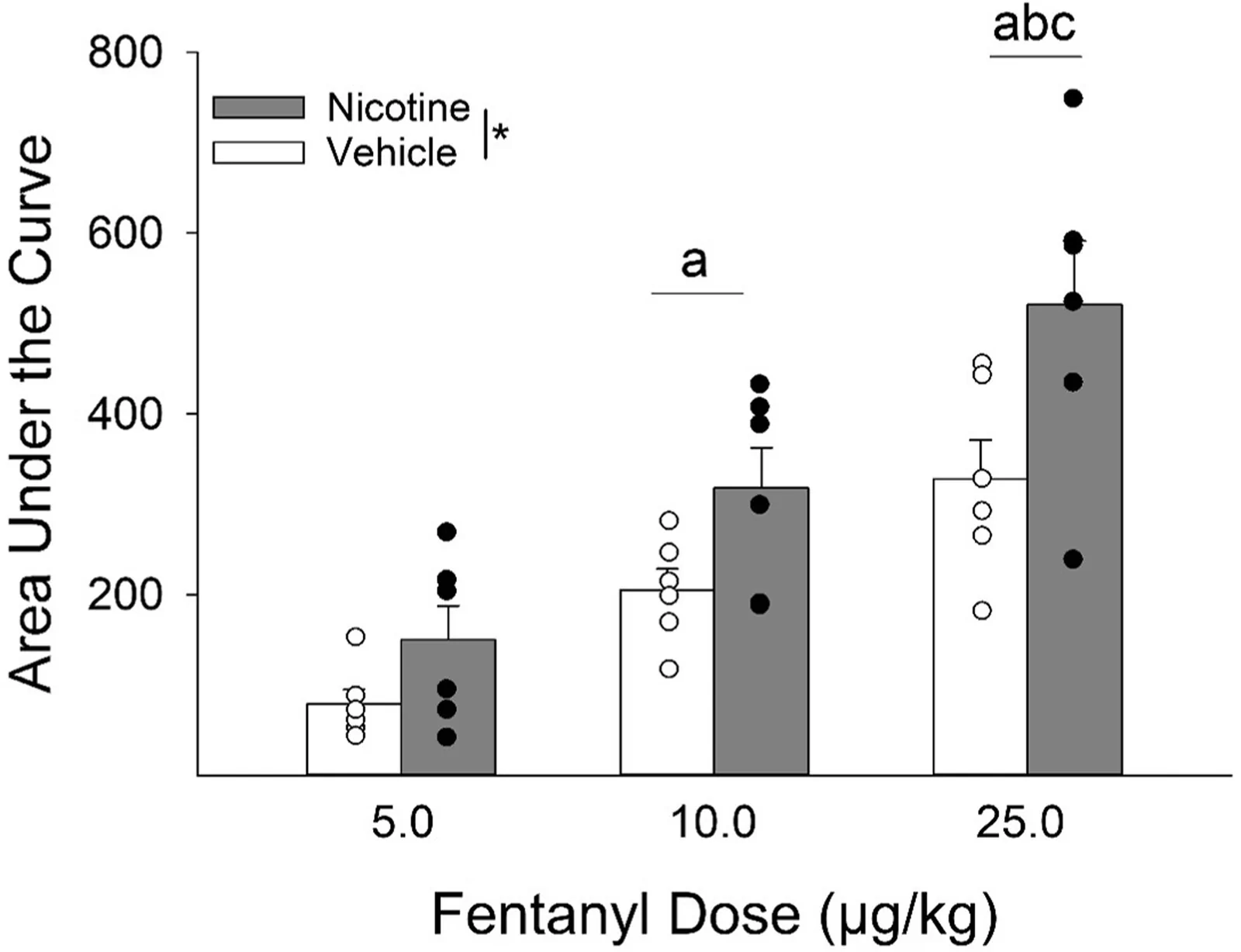
Nicotine treatment augmented concentration of DA in DLS following administration of fentanyl. AUC values derived from overall DA concentration in DLS over the recording period (∼20 min) following fentanyl injection of 5, 10, and 25 μg/kg, i.v., escalating cumulative doses. Tukey's post hoc comparisons revealed that administration of 10 and 25 µg/kg of fentanyl significantly increased the AUC compared with administration of 5 µg/kg, regardless of nicotine or vehicle treatment (a indicates *p*s < 0.001) and administration of 25 µg/kg fentanyl significantly increased the AUC above that of treatment with 10 µg/kg fentanyl, again regardless of nicotine or vehicle treatment (b indicates *p* < 0.001). Pretreatment with nicotine generally potentiated the fentanyl-enhanced DA concentration in DLS relative to vehicle pretreatment across all fentanyl doses (* indicates a significant effect of nicotine treatment, *p*s < 0.05). Tukey's corrections for multiple comparisons conducted on the AUC of each dose of fentanyl between nicotine- and vehicle-treated rats (*n* = 6) revealed that nicotine pretreatment significantly enhanced DA concentration, compared with vehicle pretreatment, specifically at the 25 µg/kg fentanyl dose (*c* indicates *p* < 0.05) while the effect at the 5 and 10 µg/kg doses failed to survive correction for multiple comparisons. Extended Data Figure 6-1 depicts statistical tables for these analyses.

10.1523/ENEURO.0161-26.2026.f6-1 Figure 6-1 Statistical analyses of the AUCs obtained during the FSCV procedures following infusion of various doses of fentanyl as depicted in Figure 6. Download Figure 6-1, TIF file.

## Discussion

Administration of nicotine enhances opioid consumption and promotes inflexible-like seeking behaviors such as escalated responding for opioids that persists despite administration of contingent punishment (Cardenas et al., 2021; Loney et al., 2021; Honeycutt et al., 2022; Honeycutt et al., 2024a; Honeycutt et al., 2025). Nicotine administration reliably elevates responding for opioids relative to nicotine-naive/abstinent control groups as evidenced by both clinical (Frosch et al., 2000; Nahvi et al., 2006; Plesner et al., 2016; Weinberger et al., 2017; Lichenstein et al., 2019; Parker and Weinberger, 2021; Wang et al., 2021) and preclinical studies (Cardenas et al., 2021; Loney et al., 2021; Honeycutt et al., 2022; Honeycutt et al., 2024a,b; Honeycutt et al., 2025). Despite numerous behavioral studies, the precise neurochemical mechanisms driving this faciliatory effect of nicotine are not fully understood. It is thought that the emergence of late-stage addictive-like behavioral phenotypes is associated with a shift in DA-mediated control of behavior from the VMS to DLS (Everitt and Robbins, 2005, 2013, 2016). As such, the present study sought to further characterize the behavioral and neurochemical effects of the interactions between nicotine and fentanyl, including examining the effect of nicotine on fentanyl-induced DLS DA transmission. The first experiment aimed to determine whether treatment with nicotine increases consumption of fentanyl, in part, through altering sensitivity to the effects of fentanyl by applying a dose–response curve procedure to measure IVSA of fentanyl as a function of fentanyl dose. The second experiment served to establish the degree to which nicotine affects fentanyl-evoked DA transmission in DLS as measured by FSCV.

We first employed a behavioral dose–response curve to test the effect of pretreatment with nicotine (0.4 mg/kg, s.c.) or vehicle in controls on the pattern of behavioral responding to obtain opioids as a function of varying fentanyl doses (0.75, 1.5, 3.5, and 5.0 μg/kg/inf) in order to determine if nicotine administration altered the behavioral sensitivity to fentanyl as a function of dose. We observed that rats treated with nicotine prior to operant sessions consumed significantly more fentanyl relative to vehicle-treated controls across the initial acquisition phase (Fig. 1*A*). Analysis of the percentage of active lever responses relative to total (active and inactive) lever responses revealed that nicotine-treated rats made more active responses and acquired drug-directed responding sooner compared with control rats (Fig. 2*A*). Specifically, by the second day of acquisition, nicotine-treated rats had reached an average accuracy of ∼85% in active responding, whereas the controls did not reach 85% accuracy until the seventh day of acquisition. Importantly, all rats did develop fentanyl-directed responding by the end of the acquisition phase before they were advanced to the dose–response portion of the experiment (Fig. 2*B*). Additionally, during acquisition, female rats in general, regardless of nicotine or vehicle condition, consumed more fentanyl and demonstrated higher active response accuracy relative to male rats. This finding is not unsurprising, as it has previously been shown that female rats will acquire opioid self-administration more rapidly and consume more opioids relative to male counterparts (Bertz et al., 2016; Thorpe et al., 2020; Towers et al., 2022). Importantly, within the present study, the observed effect of sex on drug-directed responding did not produce statistical interactions with nicotine treatment, further indicating that the effect of nicotine to promote acquisition of responding for fentanyl does not differ between sexes. Thus, despite these differences between male and female rats in fentanyl consumption, nicotine promoted enhanced responding for fentanyl equally between sexes relative to vehicle-treated controls.

Following acquisition of operant responding for fentanyl at the 1.5 μg/kg/inf dose, trained rats were exposed to various doses of fentanyl in a semirandom order (order of presentation counterbalanced across experimental conditions) to assess the effect of nicotine on responding for the consumption of fentanyl relative to control rats. We found that across all tested doses of fentanyl (0.75, 1.5, 3.5, and 5.0 μg/kg/inf), nicotine treatment (0.4 mg/kg, s.c.) resulted in elevated consumption of fentanyl relative to rats treated with vehicle (Fig. 3), indicative of a vertical shift in the dose–response curve. This finding of a vertical shift in responding across all doses of fentanyl demonstrates a behavioral phenotype that has often been associated with vulnerability for the development of drug addiction (Piazza et al., 2000) and further supports that concurrent nicotine administration promotes the liability for misuse of opioids. This interpretation is founded on previous work that has shown that subjects demonstrating vertically shifted responding in a dose–response curve will also display other addictive-like behaviors (e.g., high responsivity to novelty, reduced threshold dose necessary to acquire drug self-administration, etc.) and would be prone to drug consumption in a naturalistic environment offering various drug doses as opposed to an experimenter-controlled fixed dose (Piazza et al., 2000), suggesting that vertical shifts in the dose–response curve for drug consumption have predictive value for identifying particularly vulnerable phenotypes. As such, this finding suggests that nicotine is driving augmented responding for opioids by enhancing motivation to consume opioids regardless of dose. Alternatively, had rats across nicotine and saline conditions been observed to collapse in responding for fentanyl at either lower or higher doses, resulting in a respective leftward or rightward horizontal shift in the dose–response curve, such a behavioral pattern could indicate that nicotine alters sensitivity to fentanyl. As demonstrated here with fentanyl citrate, nicotine treatment augments motivation to consume opioids likely without affecting sensitivity to the effects of fentanyl. This interpretation is also supported by findings from behavioral economics procedures applying a within-session dose–response curve to produce indices of motivation, demonstrating that nicotine elevates motivation to obtain the opioid remifentanil (Honeycutt et al., 2025). In summary, nicotine produces a drug-vulnerable behavioral phenotype in responding for fentanyl, and these effects do not appear to be due to any nonspecific effects of nicotine to alter the overall sensitivity to fentanyl. Moreover, these results provide support for using equivalent doses between nicotine-treated and controls rats in measuring differences in the neurochemical responses to fentanyl as we have done in the FSCV procedures discussed below.

Admittedly, the fentanyl dose–response curve presented here utilized an abbreviated range of doses; therefore, it did not result in a complete inverted U-shaped distribution of responses. The dose of 1.5 μg/kg/inf of fentanyl was selected for training acquisition of IVSA and for interim sessions between presentations of varied doses to effectively reinforce operant responding within short-access sessions. Had lower doses of fentanyl (<0.75 μg/kg/inf) been included in the curve procedures, a complete inverted U would likely have emerged. However, doses substantially below the trained dose could likely interfere with continued responding, potentially confounding interpretation of behavioral responses within the dose–response curve, and were therefore not included in this study. While the exclusion of doses of fentanyl low enough to produce a complete inverted U-shaped curve is a limitation of the present study, the doses tested here strongly support that nicotine induces a vertical shift in responding for fentanyl.

An additional consideration in the interpretation of the vertical shift being indicative of enhanced motivation for opioids is the known effect of nicotine to promote responsivity to drug-associated cues and contextual stimuli. Nicotine administration enhances approach to cues associated with the reinforcing properties of drugs (Bevins and Palmatier, 2004; Maddux and Chaudhri, 2017; Stringfield et al., 2018; Loney et al., 2019) and limits avoidance of cues associated with aversive properties of drugs (Bienkowskia et al., 1998; Kunin et al., 1999; Araki et al., 2004; Ishida et al., 2011; Rinker et al., 2011; Loney et al., 2019; Loney and Meyer, 2019), and as such, the use of cue lights, retractable levers, and the contextual cues of the operant chambers likely contribute to nicotine-enhanced responding within these procedures. Therefore, experimental parameters excluding such external stimuli associated with receiving fentanyl may result in a reduction in the nicotine-mediated elevation in responding for opioids. While the effect of nicotine to enhance salience of drug-associated cues likely contributes to augmented responding for opioids irrespective of other neurobiological mechanisms, it is important to note that nicotine has also been shown to enhance the reinforcing value of opioids, as measured with conditioned place preference paradigms while simultaneously interfering with opioid-induced conditioned taste avoidance procedures (Shams et al., 2006; Vihavainen et al., 2008; Loney et al., 2021; Mukherjee et al., 2024), and so there are likely additive or synergistic effects between the ability for nicotine to enhance cue reactivity and augment the reinforcement of opioids on the observed escalated consumption of opioids. This calls for more parametric studies to specifically disentangle the role of heightened cue reactivity from drug reinforcement in nicotine and opioid polysubstance research.

In the second experiment, FSCV was used to measure the effect of noncontingent administration of fentanyl (cumulative dose of 25 μg/kg, i.v.) on DA transmission in nicotine-treated (0.4 mg/kg, s.c.) and vehicle-treated control rats. Infusions of fentanyl rapidly and dose-dependently increased extracellular DA concentration in the DLS (Figs. 4–6). Although FSCV is generally used to measure rapid changes in DA transmission, long-term baseline concentration changes (over 20 min) are observed under certain drugs or combinations of drugs (Bhimani et al., 2021; Pauly et al., 2023). It is noteworthy that there is a paucity of FSCV studies investigating naturally occurring DA transients in the DLS of awake-behaving rats due to their relatively low concentration (Robinson et al., 2002; Salinas et al., 2023). In addition, prior work applying FSCV to characterize DA transmission in the striatum has shown that the ratio of phasic-to-tonic-evoked DA signals is generally higher in NAc relative to DLS, suggesting that tonic DA in DLS is generally elevated relative to NAc, such that facilitated phasic DA release is less apparent in the DLS than NAc (Zhang et al., 2009a). Another study (Zhang et al., 2009b) has shown nicotine to decrease tonic DA release in both the NAc and DLS; however, phasic release is predominantly enhanced in the NAc rather than the DLS. In this study, subcutaneous nicotine alone did not elevate baseline DLS DA transmission, which is consistent with previous microdialysis studies (Johnson et al., 2000) showing that nicotine-evoked DLS DA is only slightly increased compared with NAc DA (Zhang et al., 2009b). It is noteworthy that FSCV has much higher spatial resolution than microdialysis and that such sampling procedures may include DA from other neighboring brain regions (e.g., olfactory tubercle, ventral pallidum). Therefore, the enhanced baseline DA from nicotine may either be well below the limits of detection of FSCV (Venton and Cao, 2020) or due to the specificity of recording exclusively in the DLS. However, when fentanyl (5–25 μg/kg, i.v.) was infused with nicotine on board, measured DA signals were robustly enhanced relative to the saline-pretreatment condition (Figs. 4–6). These findings demonstrate that fentanyl augments DA transmission in DLS, and pretreatment with nicotine potentiates this effect, resulting in enhanced fentanyl-induced DA transmission within DLS when nicotine is systemically administered. Additionally, nicotine-induced desensitization of nAChRs, particularly the α4β2 subtype, results in a suppression of tonic DA within both the DLS and VMS while simultaneously augmenting phasic, frequency-dependent DA release. This has been shown to be more pronounced in the dorsal striatum compared with the VMS (Chen et al., 2019), which also may contribute to the lack of an increase in baseline DA signal following nicotine administration. Furthermore, nicotine promotes DLS DA release preferentially by binding to presynaptic α4β2 nAChRs on SN DA neuronal terminals as opposed to actions on SN DA neuronal cell bodies (Partridge et al., 2002; Xiao et al., 2009; Ehlinger et al., 2017; Licheri et al., 2018). This suggests that nicotine may be acting on SN-DLS DA release by preferentially enhancing DA released in response to other salient stimuli (Leonard et al., 2025).

We observed that each cumulative fentanyl dose significantly increased DA relative to the initial 5 μg/kg dose. Given the generally elevated volume of fentanyl-induced DA transmission in DLS, our demonstration of nicotinic facilitation of fentanyl-enhanced DA in DLS is remarkable. Fentanyl increases DA transmission in the DLS, presumably through inhibition of local cholinergic interneurons in the DLS and GABAergic neurons in the VTA and SN leading to greater excitation of DA neurons (Reeves et al., 2022). Regardless of the mechanism driving fentanyl-induced increases in DLS DA concentration, nicotine was observed to substantially augment fentanyl-evoked DA across each of the doses of fentanyl in male rats; however, the precise mechanism driving this elevation of DA in DLS remains to be fully elucidated. Lastly, these FSCV experiments were conducted in male rats to demonstrate a mechanistic baseline for polysubstance use of nicotine and fentanyl. Given that the estrous cycle plays a key role in modulating dopaminergic responses to drugs of abuse (Bhimani et al., 2026), we cannot rule out the possibility of nicotine–fentanyl sex differences on DLS DA.

One possible confound in the interpretation of our finding for nicotine to enhance fentanyl-induced DA release within the DLS is that this dose of nicotine has been shown to enhance locomotor activity (Reavill and Stolerman, 1990) and thus the increase in locomotion could have influenced our DA measurements. While we did not specifically measure locomotion in this study, it should be noted that we did not observe a measurable increase in DA concentration following administration of nicotine at a time point that it would be expected to increase locomotion. This strongly suggests that differences in locomotion likely played a minimal role in the ability for nicotine to enhance fentanyl-induced DA concentrations within the DLS though it should be noted that not measuring locomotion is a limitation of the current study. An additional limitation of the current study is the use of a single nicotine dose in both the behavioral and neurochemical studies. While we chose to employ a dose of nicotine that has been well characterized across a number of prior studies, future work should focus on expanding the nicotine dose–response curve as it pertains to the ability to enhance opioid-induced behaviors and DA concentrations. Finally, both nicotine and DA signaling have been shown to have faciliatory effects on attention, associative learning, and working memory (Nieoullon, 2002; Spinelli et al., 2006; Day et al., 2007; Loney et al., 2021). As such, the ability for nicotine to enhance these factors could contribute to our observations of enhanced acquisition of fentanyl self-administration and subsequent increased fentanyl consumption. Admittedly, this was our rationale for conducting the FSCV measurements with passive administration of fentanyl as opposed to operant self-administration. Regardless, future studies should be conducted to demonstrate the role of nicotine to alter DA transmission on these cognitive components which would most certainly play an integral role in drug self-administration.

This is the first study to characterize the effects of nicotine and fentanyl on the release of DA within DLS. These findings align with prior work in opioids that have shown that DLS DA eventually becomes necessary to support heroin seeking following extended experience with heroin self-administration (Hodebourg et al., 2019), in a similar manner to what has been shown with cocaine (Takahashi et al., 2007; Belin and Everitt, 2008; Willuhn et al., 2012). The present study demonstrates a potential mechanism by which nicotine may facilitate this shift in behavioral control by potentiating DA transmission in DLS without extended training with fentanyl self-administration. Importantly, it has been previously shown that nicotine mediates DA in DLS but becomes more impactful during later stages of drug-conditioning to regulate DA responses in DLS induced by learned opioid-related cues (Zhou et al., 2001). The present work demonstrates a potentiating effect of nicotine on DLS DA in rats otherwise naive to fentanyl, without extended experience with fentanyl. This finding strongly suggests the mechanistic role of nicotine to promote escalation of, and subsequent development of inflexible responding for, opioids mediated by boosted DA activity within the DLS. This may facilitate the transition from VMS to DLS control of drug-conditioned decision-making and may underlie the enhanced misuse vulnerability observed in consumers of nicotine-containing products.

## Synthesis

Reviewing Editor: Rosemary Bagot, McGill University Decisions are customarily a result of the Reviewing Editor and the peer reviewers coming together and discussing their recommendations until a consensus is reached. When revisions are invited, a fact-based synthesis statement explaining their decision and outlining what is needed to prepare a revision will be listed below. The following reviewer(s) agreed to reveal their identity: Belle Buzzi.

N/A

## Author Response

We thank the reviewers for the thoughtful and thorough critique of our manuscript. Through addressing each of the reviewers' concerns we believe that the manuscript has been strongly improved. We outline our response to the raised concerns below:

Reviewer 1 This study provides a useful advance by demonstrating that nicotine potentiates fentanyl-induced dopamine signaling in the DLS while increasing fentanyl self-administration. However, the advance is moderate rather than transformative because the neurochemical findings remain correlational, are restricted to males, and do not directly establish a causal role for DLS dopamine in nicotine-enhanced opioid use.

The authors test if nicotine increases fentanyl self-administration and fentanyl-evoked dopamine release in the dorsolateral striatum (DLS). Using fentanyl IVSA and FSCV, they show that nicotine increases fentanyl intake and potentiates fentanyl-induced DLS dopamine release. The topic is interesting and clinically relevant given the prevalence of nicotine-opioid co-use. The behavioral and neurochemical findings are generally clear and complementary. However, several interpretations are overstated, particularly regarding causality and DLS mechanisms. The manuscript is well written but could be substantially shortened, especially the Introduction and portions of the Discussion. Overall, the study provides a moderate advance by identifying a potential DLS dopamine mechanism contributing to nicotine-enhanced opioid use.

Major concerns • The manuscript reports several sex-dependent behavioral effects, yet the figures do not allow readers to adequately evaluate these findings. Sex-disaggregated data should be provided, preferably in the main figures or, at minimum, in the Supplemental Material. Individual animals should be displayed with sex-specific symbols or colors.

This was a point that was also raised by Reviewer 2. We agree with both reviewers and have since edited the existing figures, and added additional figures, that disaggregate the data by sex and, where appropriate, have included bar graphs that depict individual data points for each subject with sex demarcated by separate symbols. • The rationale for excluding females from the FSCV experiments is not convincing. The existence of known sex differences in dopamine release provides a rationale for studying both sexes rather than restricting the neurochemical analyses to males. Consequently, the proposed mechanism cannot be generalized beyond males.

We completely agree with the reviewer that sex differences in the dopaminergic system and response to opioids are critical areas of investigation. We appreciate this constructive critique and recognize that our findings cannot be generalized to female rats at this stage.

The decision to restrict these initial, highly challenging awake-behaving FSCV experiments to male rats was driven by the extreme technical demands of combining jugular vein catheterization with awake-behaving, tethered voltammetric recordings. Given the need to first establish a robust, baseline neurochemical mechanism for fentanyl at these specific fixed doses, we focused our resources on a single sex to ensure statistical power.

We fully acknowledge this as a limitation of the current study. In accordance with the reviewer's concern, we have updated our Discussion section to explicitly state that these mechanisms are currently limited to males and to highlight how known sex differences in DLS dopamine dynamics warrant a dedicated, independent study in females in the future. Importantly, we have cited recently published data examining sex-specific/estrous cycle dependent changes in dopaminergic responses in the nucleus accumbens of rats to methamphetamine. These data demonstrate that, while the effects of methamphetamine follow a similar pattern between males and females, estrous cycle stage plays a significant role in regulating dopamine signaling, which distinctly impacts psychostimulant induced dopamine transmission. Therefore, including females in this work would require several different groups of rats at different estrous cycle stages for the study of sex differences to reach statistical significance. This is a limitation of the current work but is a goal for future studies • Relatedly, the presentation of group means alone obscures the extent of individual variability. For key behavioral outcomes, perhaps adding total fentanyl intake and overall active-lever response accuracy with individual/sex data points would improve reporting of the data and interpretation.

As addressed in point one, we have edited the figures to include individual data points for each of the most pertinent findings. These include total fentanyl consumption during acquisition (Fig 1B), active-lever accuracy on the final day of conditioning (Fig 2B), and earned infusions/fentanyl consumption during the dose response curve (Fig 3C&D) as well as for the AUCs in Fig 6. • Nicotine and dopamine is well known to enhance attention, associative learning, and working memory (with a U inverted dose-response). These cognitive effects could contribute to the faster acquisition and higher active-lever accuracy observed in nicotine-treated rats and should be discussed as alternative explanations.

Again, we agree with the reviewer, and this was actually one of the initial rationales for conducting the FSCV studies with passive fentanyl administration in untrained rodents. As such, we have edited the Discussion to include these as alternative explanations and to highlight that future studies should attempt to disentangle the effects of nicotine and dopamine on these cognitive processes.

Minor Concerns • The Introduction is overly long and contains substantial material that would be more appropriate for the Discussion. A more focused rationale would improve readability and emphasize the study's novelty.

We have edited the Introduction to be more focused and have moved some sections to the Discussion as suggested. • The discussion of hypothetical leftward and rightward shifts in the dose-response curve is unnecessarily lengthy given that neither effect was observed and the implications of the observed vertical shift are already discussed.

We have significantly reduced the discussion of the dose-response curve in an attempt to remain focused on the actual outcomes that were observed. • Figure 4 is difficult to interpret. The grouping of traces, representation of SEM, and effective sample sizes are unclear. Improving the figure and legend would facilitate interpretation of the FSCV results.

We have edited Figure 4 to make the key results easier to interpret and have likewise edited the figure legend to more accurately illustrate the key points of the traces. Specifically, Figure 4 shows average baseline DLS-dopamine concentration changes vs time (n = 6 rats/Subfigure) with either vehicle or nicotine on board 5 minutes before and 20 minutes after fentanyl administration. Average changes are binned in 60s intervals. We have modified the figure to show an injection time when IV fentanyl was administered as well as clarified the "y-axis" is dopamine concentration as represented by the scale bar.

Reviewer 2 This manuscript explores the effect of nicotine (0.4 mg/kg) on fentanyl IVSA behaviors. Additionally, the authors use FSCV to measure dorsal lateral striatum dopaminergic transmission in response to fentanyl and/or nicotine + fentanyl. They found that nicotine enhanced fentanyl intake and that nicotine enhanced the fentanyl mediated DA response in the DLS. While the manuscript is interesting and adds a novel mechanism, there are several changes that are recommended to improve their manuscript.

General comments:

- Given that this dose of nicotine is listed as a standard dose used in behavioral studies (line 155), the mention of effect on locomotor activity should be mentioned as to not be considered a potential confounding factor.

We agree with the reviewer that this should be listed as a potential confounding factor and have amended the Discussion to include this as a possible confound.

- Given the main effects of sex, it will be helpful to show both sexes separately either in the main manuscript or supplemental material.

As outlined above, we have now included edited figures in the manuscript and new figures in the supplementary materials that illustrate the relevant outcomes disaggregated by sex - Given the use of a single dose of nicotine, and the narrow range of rewarding doses of nicotine in rodents depending on age and potentially sex (PMID: 25414778), discuss possible implications for dose responsiveness of your findings. Add the use of a single dose of nicotine is a limitation of the study.

Again, we agree with the reviewer that this is a limitation of our current design and have included such in the discussion and specifically called for future studies to provide a more robust nicotine dose-response curve with regards to our current findings.

- Provide the Ns in the Methods and figure legend.

The subject numbers for each experiment have been added to the Methods and each figure legend.

- All post-hoc tests should be named either in methods or in results.

This was inadvertently left out of the original manuscript. We have now indicated in the Data Analyses section that Tukey's corrections for multiple comparisons was used for all post-hoc analyses.

Line by line comments:

Line 114: "For FSCV procedures only male subjects were included (n = 9; Charles River, Wilmington, MA) to avoid potentially confounding effects of sexual dimorphisms in DA release and uptake in FSCV known to occur between male and female rats (Walker et al., 1999). " Please discuss this further in the discussion section and include implications from literature on sex hormone related effects on differences in dopamine release for both fentanyl and nicotine. In fact, sexual dimorphism is the main reason for including rodents of both sexes. Add the use of male only as a limitation of the study.

As mentioned above (response to reviewer 1), the goal of this study was to show that nicotine potentiates fentanyl-enhanced dopamine transmission in the DLS. We have emphasized in the discussion that while this study does not take sex differences into account at the neurochemical level, it does imply a role for nicotine enhancing the effects of opioids such as fentanyl.

Figure 4: what is the Y axis? Is this one rat? As reviewer 1 suggested we have clarified this in the figure and legend. Please see the comment above.

Specifically, Figure 4 shows average baseline DLS-dopamine concentration changes vs time (n = 6 rats/figure) with either vehicle or nicotine on board 5 minutes before and 20 minutes after fentanyl administration. We have modified the figure to show an injection time when IV fentanyl was administered as well as clarified the "y-axis" is dopamine concentration as represented by the scale bar.

Line 262: Is there a session x nicotine interaction? There was no session x nicotine interaction. We have now included stats tables in the Supplementary Materials which list all statistical outcomes for all experiments.

Line 292: significant Nicotine X Dose X Sex interaction was observed (F(3, 51) = 3.40, P < 0.05), and post-hoc analyses revealed this effect was driven by nicotine-treated female rats earning more infusions specifically at the lowest dose of fentanyl (0.75 μg/kg) relative to all other experimental groups.

List the post-hoc test that was used and report the p -values in the text and in the figure.

The post-hoc tests, p-values, and subject numbers have been added to the text and the figure legends.

Line 299: and post-hoc analyses revealed that this effect was driven by female rats consuming more fentanyl than males specifically at the two highest doses of fentanyl (3.5 and 5.0 µg/kg/inf; data not shown).

It would be useful to show these data, especially the breakdown by pretreatment.

As outlined above, we now include additional figures to demonstrate individual data points and disaggregated by sex.

Line 301: Importantly, no Nicotine X Dose interaction was observed, demonstrating that nicotine treatment did not differentially alter consumption of fentanyl at any particular dose relative to vehicle treatment.

Does this mean that posthoc comparisons did not reveal a significant difference between nicotine pretreated and vehicle pretreated rats in terms of fentanyl infusions? The rest of the paragraph infers otherwise and post-hoc data here at all doses of fentanyl may be useful for readers. I suggest making a table if possible.

We apologize for the confusion. This passage is specifically referring to the statistical analyses of fentanyl consumption. We did observe a statistical interaction between Sex, Nicotine, and Dose for the number of earned infusions, but when specifically analyzing consumption this result was not upheld. Regardless, we have now included complete statistical tables for all analyses in the Supplementary Materials.

Line 309: Representative DA concentration vs time traces at the oxidation potential for DA (~0.65V) in rats with saline or nicotine on board followed by fentanyl injection (5 μg/kg, IV) show that noncontingent delivery of fentanyl produced a rapid increase in concentration of DA in DLS, and this effect was augmented by treatment with nicotine (Fig. 3);

Please give a rationale for use of 5 ug/kg IV fentanyl. It is unclear from results whether nicotine pretreatment increases earned infusions of fentanyl in figure 2A at that dose.

We have clarified this in text. Briefly, this dose of fentanyl was selected because it is the lowest reinforcing dose of fentanyl in behavioral studies (e.g. conditioned place preference). Due to individual variability in the number of presses rats take in our study, we selected a "low," "medium," and "high" dose of cumulative fentanyl taken to compare neurochemical responses while still maintaining statistical power.

Line 313: Figure 4 shows average changes in DA concentration every 60 seconds 5 minutes before and 20 minutes after fentanyl administration in vehicle and nicotine treated rats.

Please specify the average of how many rats were used for these images.

Again, this has been clarified in the text and figure the n values.

Line 319: Post-hoc analyses determined that 10 μg/kg significantly increased DA concentration relative to 5 μg/kg and that 25 µg/kg significantly increased above the 10 µg/kg dose (Fig 5).

Please list post-hoc analyses used and show significance mentioned, if possible, on the figure for a better understanding.

The figure has been updated to display all of the significant outcomes and the figure legend has been edited to indicate the post-hoc and significance level.

Figure 4 legend appears blurry. If it is possible to have a higher resolution image, please replace.

We changed the picture format to have a higher resolution without pixilation.

Line 455: Subcutaneous nicotine alone did not elevate baseline DLS DA transmission Please discuss this further with overall implication of the fact that nicotine was not taken volitionally and how this could have potentially impacted all findings, especially the findings on DA.

Subcutaneous nicotine did not significantly elevate baseline DLS dopamine transmission as evidenced by FSCV in our study. This is not to say that nicotine does not elevate dopamine as previous studies have shown that nicotine does in fact increase "phasic" dopamine transmission especially in the NAc but not tonic DA release in the DLS or NAc. (Zhang et al., 2009). However, microdialysis studies have shown that nicotine enhances baseline dopamine concentration in the dorsal striatum to a much smaller extent (~20%) than the nucleus accumbens or no increase, specifically, dorso "lateral" striatum (Pontieri &Di Chiara 1999). Additionally, previous electrophysiology and voltammetry studies (Chen 2019 and Zhang 2009) have shown that nicotine inhibits "tonic"(a lower baseline firing rate <8Hz) dopamine release and its inhibition is much more effective on phasic release in the dorsolateral striatum (DLS). Therefore, due to decrease in nicotine-induced tonic DA release, electrode drift, and background subtraction in FSCV, these small DA increases from phasic-bursts may be masked or under the limit of detection. We have emphasized this in text and added additional references supporting this. In regard to the second point that nicotine was not taken volitionally; while nicotine did not increase baseline dopamine in this study, it did not decrease it either. FSCV can be used to study both relative increases and decreases in catecholamine concentration. Given that we did not observe any decrease in baseline dopamine and control rats were given vehicle injections, as well as the rapid onset of baseline dopamine increase following fentanyl administration, we do not see this as an issue to impact findings.

Line 497: This finding strongly suggests a mechanistic role for nicotine to promote escalation of, and subsequent development of inflexible responding for, opioids mediated by boosted DA activity within the DLS, which may facilitate the transition from VMS to DLS control of drug-conditioned decision making and may underlie the enhanced misuse vulnerability observed in consumers of nicotine-containing products.

This is a long and convoluted sentence. It is recommended to split into two.

This has been split into two sentences as the reviewer suggested.
